# Hydrocephalus associated to posterior fossa tumor-like sarcoidosis: A case report and literature review

**DOI:** 10.22088/cjim.13.3.646

**Published:** 2022

**Authors:** Ghassen Gader, Chaden Fakhfakh, Alia Zehani, Sabeur Thamlaoui, Ihsèn Zammel, Mouna Rkhami, Mohamed Badri

**Affiliations:** 1 Department of Neurosurgery, Trauma and Burns Center, Ben Arous, Tunisia; 2 Department of pathology, La Rabta hospital, Tunis, Tunisia; 3 Department of anesthaesiology, National Institute of Neurology, Tunis, Tunisia

**Keywords:** Inflammatory Pseudotumour, Sarcoidosis, Neurosurgery, Internal Medicine

## Abstract

**Background:**

Intracranial inflammatory pseudotumors (IIPT) are one of the differential diagnosis for the central nervous system (CNS) tumors. They represent a rare condition that may mimic clinically and radiologically intracranial tumors and induce their complications. Among their etiologies, neurosarcoidosis is one of the less known and less frequent. To the best of our knowledge, only two cases of posterior fossa IIPT have been reported in the literature. We present here the 3rd case related to a neurosarcoidosis.

**Case presentation:**

We report the case of a 55-year-old female patient who presented with an altered state of consciousness associated to severe intracranial hypertension syndrome for four months. Glasgow coma scale on admission was 14/15. Brain imaging revealed bilateral cerebellar micronodular meningeal enhancement regarding the mesencephalon and the pons, as well as a nodular lesion of the 4th ventricle causing a triventricular acute hydrocephalus. The patient had a ventriculo-peritoneal shunt with a favorable outcome. Afterwards, she underwent a salivary gland biopsy which confirmed the diagnosis of neurosarcoidosis.

**Conclusion:**

Posterior fossa IIPT are very rare, mainly when located in the posterior fossa, leading to confusion with other pathologies. MRI has an important role in the diagnosis of these lesions, and the determination of their etiology. It shows other than the IIPT itself, many other signs such as leptomeningeal enhancement, nodular lesions or pituitary stalk thickening. These signs can orientate towards the diagnosis. Treatment may associate to symptomatic approach, corticosteroids. Surgical resection may be proposed when the diagnosis remains doubtful.

Intracranial inflammatory pseudotumors (IIPT) are one of the differential diagnosis for the central neurologic system (CNS) tumors. They represent a rare condition that can mimic clinically and radiologically intracranial tumors and induce their complications (1,2). Most IIPT arise from the dural and meningeal structures (2). They remain an unclear etiopathogeny. On the other hand, sarcoidosis is a chronic granulomatous disease of an unknown origin, characterized by noncaseating granuloma which may affect any organ, including the CNS (3). To the best of our knowledge, only two cases of posterior fossa IIPT have been reported in the literature (1). We present here the 3^rd^ case, which was related to a neurosarcoidosis. 

## Case description

A 55-year-old female patient with a history of hypertension and type 2 diabetes mellitus presented at our hospital emergency with an altered state of consciousness associated to severe intracranial hypertension syndrome made of headaches and vomiting. 

**Figure 1:**
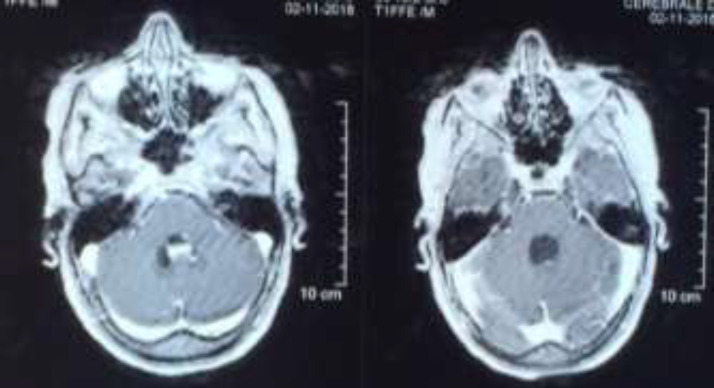
Axial section of a brain MRI on T1-weighted image with contrast injection revealing a cystic lesion of the 4th ventricle, associated to a nodular enhancement of the central and lateral apertures and of the fifth cranial pair

**Figure 2:**
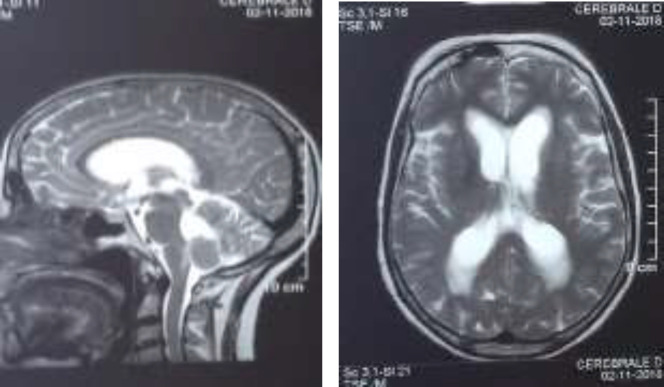
Axial (A) and Sagittal (B) sections of a brain MRI on T2-weighted imaging showing acute tri-ventricular hydrocephalus.Figure B can also notice the lesion in the 4th ventricle causing the hydrocephalus

Occipital headaches, vomiting, visual blur and vertigo were noticed in the past four months without any history of head trauma or fever.On admission, her Glasgow coma scale was 14/15. She had no motor weakness. Brain magnetic resonance imaging (MRI) revealed bilateral cerebellar micronodular meningeal enhancement (figures 1 and 2). This enhancement concerns also the peduncular and protuberentialsubarachnoidal space and the fifth cranial pair. This exam also showed a pituitary stalk thickening and a nodular lesion of the 4th ventricle causing a triventricular dilatation with a nodular enhancement of the central and lateral apertures. 

Preoperative assets were in favor of a posterior fossa granulomatosis. And due to the acute hydrocephalus, a ventriculo-peritoneal shunt was performed.

Postoperative, the patient had no more intracranial hypertention syndrome.

A sputum stain for mycobacterium, the Mantoux test and culture isolation of mycobacteria were negative, as well as the cerebrospinal fluid assesment and angiotensin enzyme assay which were normal. The patient was discharged one week after surgery and was referred to the internal medicine department. 

**Figure 3:**
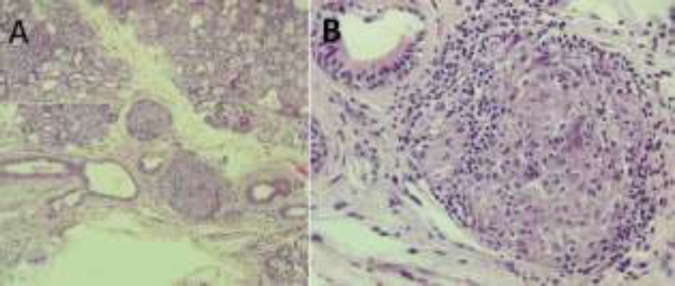
(A) Hematoxylin Eosin X10 showing a salivary gland containing granulomatous lesions. (B) Hematoxylin Eosin x 40 showing an epithelioid granuloma without necrosis surrounded by lymphocytes

**Figure 4:**
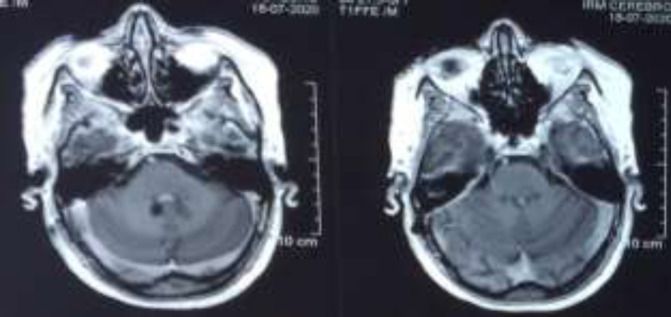
MRI findings after glucocorticoids-based treatment

Thereby, she underwent a salivary gland biopsy (figure 3) which confirmed the diagnosis of neurosarcoidosis. This motivated a long-term treatement based on corticosteroids. Six months after, the patient had a control MRI showing a complete regression of the cystic lesion, and persistence of the multiple micronodular enhancements of the subarachnoidal spaces and the patchy aspect of the basal ganglia (figure 4). 

## Discussion

IIPTs are a rare entity, described as a reactive, inflammatory and non-neoplastic phenomenon that can occur in any area in the human body, but mainly involves lungs and orbit (3). Posterior fossa IIPTs are extremely rare. A literature review made by Yu-Jun Lin et al. showed only two previous case reports of such tumors (1). We proceeded through a pubmed research using the MeSH terms «Posterior fossa» and «Inflammatory Pseudotumour». We also found only 2 concordant cases summarized on table n°1.

**Table 1:** Summary of the results found on the litterature

**Authors**	**Age/Sex**	**Location**	**Clinical**	**Surgery / treatment**	**Follow-up**
Jung et al.	52 / M	Left cerebellopontine angle	Headache and facial numbness	Surgery: near total resection of the tumor	No evidence of recurrence in 18 months
Lin et al.	49 / M	Left cerebellopontine angle	Dizziness, hoarseness, hiccup, unsteady gait	Surgery: En bloc resection of the tumor	Local recurrence at six months, no evidence of recurrence in 2 years after radiotherapy
Present case	55 / F	Fourth ventricle	Altered state of consciousness, headache, vomiting, visual blur, vertigo,	Surgery: ventriculoperitoneal shunt for the treatment of hydrocephalus -Treatment: glucocorticoids	Complete regression after one year of glucocorticoids

The pathogenesis of inflammatory pseudotumors remains unclear (1). Histologically, IIPT contains cells associated with both acute and chronic inflammatory process including lymphocytes and plasma cells, myofibroblastic spindle cells as well as a fibrosis reaction. It may be caused by trauma, postoperative inflammation, infection (EBV and HSV 8), low grade fibrosarcoma with inflammatory cells, immunological disorders and chronic granulomatous disease such as sarcoidosis(4). IIPT mostly arise from the dura mater. They are often mistaken as meningiomas with lymphoplasmacytic infiltration (1,3). Many infectious and inflammatory diseases can be the origin of these intracranial pseudotumors. Sarcoidosis is among one of the rarest etiologies.

Sarcoidosis is a chronic granulomatous disease of an unknown origin, characterized by noncaseating granuloma (5,6). This condition can affect any organ including the CNS. Neurosarcoidosis has a prevalence of neurologic involvement ranging between 3% and 10% (7,8). The diagnosis of sarcoidosis is orientated by neurologic abnormalities in 70% to 80% of the cases (6, 9). Nearly half of the patients diagnosed with neurosarcoidosis present an intraparenchymal brain lesion on imaging studies (10). These lesions may cause seizures, headaches, cognitive or behavioral problems (7, 8, 10). It can be associated with encephalopathy, vasculopathy or hydrocephalus (1). IIPT caused by sarcoidosis can be seen in any part of the CNS. However, posterior fossa locations are seldom (8, 11). In our case, the patient presented with hydrocephalus and severe increased intracranial pressure that needed an emergent ventriculoperitoneal shunt. This was related to a nodule developed inside the 4th ventricle, whose radiologic features were nonspecific. This aspect of an enhancing nodular intraparenchymal may mass lead to confusion and differential diagnosis of tumors of the same location (12). 15% to 70% of the patients with neurosarcoidosis present leptomeningeal involvement with a nodular enhancement on MRI which concerns mainly suprasellar (especially an enhancement around the hypothalamus associated with a pituitary stalk) and frontal basal meninges (13, 14). Cranial nerves neuropathy may also be encountered. They present in MRI as an enhancement of the affected nerve on contrast-enhanced T1-weighted images (4, 12). In our case, the fifth cranial nerve was affected with an enhancement as described above.

Hydrocephalus, communicating or noncommunicating, may be seen as a complication of meningeal inflammation (15). However, in our case, hydrocephalus was a result of an obstruction of the 4^th^ventricule. Cerebrospinal fluid assessment can show high protein content, pleocytosis and rarely low glucose concentration (11, 16). This profile of cerebrospinal fluid is not specific for sarcoidosis as it may be found in multiple other etiologies such as infectious meningitis or inflammatory diseases of the CNS (17). Angiotensin-converting enzyme (ACE) levels are not specific for neurosarcoidosis. Only 33% to 50% patients who had elevated ACE levels had neurosarcoidosis, thus a poor contribution to the diagnosis is associated to high level of false positive rate (18). The treatment in our case is associated to a ventriculoperitoneal shunt to resolve the life-threatening obstructive hydrocephalus, then glucocorticoids with an initial dose of 1mg/kg/day. The treatment in our case lead to a complete regression of the tumor with no evidence of recurrence after one year.

Our approach was different from those found on the litterature, which were based on surgical resection of the tumor (1). In fact, in previously reported cases, lesions were of a more important volume, and more accessible to surgery. In our case, the nodule was deep, in contact with the floor of the 4^th ^ventricle. Thus, its surgical resection was more difficult and dangerous. We solved at first the problem of hydrocephalus with perspective of treating the underlying lesion. Afterwards, surgical approach was laid off after discovering the inflammatory nature of the tumour.

The prognosis of the IIPT depends on the etiology and the site of the tumor. Even though they are considered as benign entity, they can be aggressive locally or have distant dissemination (1, 8).

In conclusions inflammatory pseudotumors remain one of the most uncommon differential diagnosis of the central nervous system tumors. Among their etiologies, neurosarcoidosis is one of the less known and less frequent, mainly when located in the posterior fossa, leading to confusion with other pathologies. MRI has an important role in the diagnosis of these lesions, and the determination of their etiology. It shows other than the IIPT itself, there are many other signs such as leptomeningeal enhancement, nodular lesions or pituitary stalk thickening. These signs can orientate towards the diagnosis. Treatment may associate to symptomatic approach, and corticosteroids. Surgical resection may be proposed when the diagnosis remains doubtful.
